# The Characteristics of Intelligence Profile and Eye Gaze in Facial Emotion Recognition in Mild and Moderate Preschoolers With Autism Spectrum Disorder

**DOI:** 10.3389/fpsyt.2019.00402

**Published:** 2019-06-19

**Authors:** Yuying He, Qi Su, Lan Wang, Wenxiang He, Chuanxue Tan, Haiqing Zhang, Manwa L. Ng, Nan Yan, Yanni Chen

**Affiliations:** ^1^Department of Pediatrics, Xi’an Jiaotong University Health Science Center, Xi’an, China; ^2^CAS Key Laboratory of Human-Machine Intelligence-Synergy Systems, Shenzhen Institutes of Advanced Technology, Chinese Academy of Sciences, Shenzhen, China; ^3^Child Healthcare Department, Xi’an Maternal and Child Health Hospital, Xi’an, China; ^4^Guangdong Provincial Key Laboratory of Robotics and Intelligent System, Shenzhen Institutes of Advanced Technology, Chinese Academy of Sciences, Shenzhen, China; ^5^Department of Pediatrics, Shaanxi University of Chinese Medicine, Xianyang, China; ^6^Child Healthcare Department, Xi’an Children’s Hospital, Xi’an, China; ^7^Speech Science Laboratory, Division of Speech and Hearing Sciences, University of Hong Kong, Hong Kong, China

**Keywords:** autism spectrum disorder, mental development, emotion recognition, eye tracking, the Griffiths Mental Development Scales

## Abstract

Childhood autism spectrum disorder (ASD) can easily be misdiagnosed, due to the nonspecific social and communicational deficits associated with the disorder. The present study attempted to profile the mental development and visual attention toward emotion among preschool children with mild or moderate ASD who were attending mainstream kindergartens. A total of 21 children (17 boys and 4 girls) diagnosed with mild or moderate ASD selected from 5,178 kindergarteners from the Xi’an city were recruited. Another group of 21 typically developing (TD) children who were matched with age, gender, and class served as controls. All children were assessed using the Griffiths Mental Development Scales–Chinese (GDS-C), and their social visual attention was assessed during watching 20 ecologically valid film scenes by using eye tracking technique. The results showed that ASD children had lower mental development scores in the Locomotor, Personal-Social, Language, Performance, and Practical Reasoning subscales than the TD peers. Moreover, deficits in recognizing emotions from facial expressions based on naturalistic scene stimuli with voice were found for ASD children. The deficits were significantly correlated with their ability in social interaction and development quotient in ASD group. ASD children showed atypical eye-gaze pattern when compared to TD children during facial emotion expression task. Children with ASD had reduced visual attention to facial emotion expression, especially for the eye region. The findings confirmed the deficits of ASD children in real life multimodal of emotion recognition, and their atypical eye-gaze pattern for emotion recognition. Parents and teachers of children with mild or moderate ASD should make informed educational decisions according to their level of mental development. In addition, eye tracking technique might clinically help provide evidence diagnosing children with mild or moderate ASD.

## Introduction

Autism spectrum disorder (ASD) is a neurodevelopmental disorder which is characterized by pervasive social communication and social interaction deficits, as well as restricted interests and/or repetitive behavior (1). A number of children with mild or moderate ASD attend mainstream kindergartens with their healthy peers. However, failing to recognize the hyperactivity, inattention, noncompliance with instructions, and repetitive and inappropriate behavior associated with ASD, these preschoolers are often labeled as “problem children” (2). As such, their teachers and parents often adopt preaching or corporal punishment as an attempt to correct their behavior. However, this often fails and results in and volatile and aggressive emotion, and sometimes violent behavior in these children. Understanding how they are different from others could greatly help teachers and parents better them and accommodate and educate ASD children. In particular, after knowing the clinical differences between mild/moderate ASD kids from their healthy peers, teachers or parents could comprehend them better and adopt a more appropriate approach to cope with their behavior problem.

Intellectual disability is a common deficit associated with ASD, and it is considered an important predictor of intervention (3–6). Examining the intelligence profile of children with mild or moderate ASD can significantly support the accurate clinical identification of the disease and design of an effective intervention regime. Intelligence tests such as Gesell Developmental Diagnosis Schedule and the Chinese Wechsler Young Children Scale of Intelligence (C-WYCSI) are commonly used in China. However, Gesell Developmental Diagnosis Schedule was revised for the Chinese population in 1981 based on an earlier version established in 1974 which was designed for children from 4 weeks to 3 years (7). C-WYCSI was made available in China in 1986 based on the 1967 version, which was used for children of 4 to 6 years old (8). Both assessments are apparently outdated and appear not suitable for preschoolers of 3–7 years old who are attending kindergarten. The Griffiths Mental Development Scales (GDS) are used to assess the development of children from birth to 8 years and is currently widely used worldwide (9). While GDS mainly focus on the social cognitive development of the child, it taps into all major aspects of a child’s development, including the physical, cognitive, social, and emotional aspects (10). The Griffiths Mental Development Scales–Chinese (GDS-C) is based on the 2006 version and revised for the Chinese population in 2016. Since then, GDS-C has been proven to be an accurate and effective test to assess the development of young children (11). Moreover, the GDS-C is also a useful assessment for evaluating the development deficits for neurodevelopment disorders involved with ASD children.

In addition to mental development, difficulty in understanding others’ emotional and mental states is considered as a core characteristic of ASD, and it is recognized as part of their social communicative impairments (1). Cognitive, behavioral, and neuroimaging studies have reported that individuals with ASD tend to have difficulties in emotion recognition across different sensory modalities (12–14). Recognition of others’ emotions and mental states relies on the processing of different emotional cues, such as facial expression, vocal intonation, body language, content of verbalization, and the complex integration of them in a dynamic context (15, 16). In TD children, emotion recognition emerges gradually throughout childhood and becomes more accurate and efficient with time. With the ability of expression recognition continuing to develop, TD children gradually become “emotion detection experts,” relying on facial emotion recognition by the age of 3–5 years (16). However, the development of emotion recognition ability is hampered in children with ASD, and ASD children have impaired discrimination and recognition of facial features, and they tend to use atypical strategies for processing facial characteristics (14, 17). Recent research findings revealed that difficulty in emotion recognition by individuals with ASD is cross-cultural, indicating the universality of facial emotion recognition deficit in this population (15).

Seeing the importance of the ability to understand other’s emotion expression in social interaction and communication, the majority of research on emotion recognition among ASD children focused on examination of facial expression during social interaction. However, many of them reported inconsistent findings (13). The inconsistencies may have originated from discrepancies in participant demographics (such as age, intelligence, ability level, and subtype), emotion type (basic or complex), and task stimuli (static or dynamic) (18). Primarily, heterogeneity and different ASD severity levels may have contributed to the varied findings. For example, low-functioning ASD children predominately showed deficits in basic emotion expression (19–22). In contrast, some studies reported no difficulties in high-functioning ASD individuals when performing facial emotion recognition tasks (23). In fact, few studies focused on children with mild or moderate ASD in mainstream educational setting (24), despite the fact that the importance of these studies as children with mild-to-moderate ASD are prone to be misdiagnosed due to the associated diversity of clinical manifestations, as well as the nonspecific manifestations of social communication during preschool age.

In addition to the heterogeneity of ASD, the type of experimental stimuli also played a determining role in studying emotion recognition in ASD children. Although there have been extensive research examining emotion recognition through facial expression in children with ASD, most relied on static photographs of different facial expressions as visual stimuli (25–27), but rarely made use of dynamic video stimuli (28–31), or ecologically valid film scenes (32, 33). Static pictures of facial expressions may not be ecologically valid (34, 35), as the details of spontaneous communication that occur during real-life social interaction may not be well captured (28). Meanwhile, real-life facial expressions are usually more subtly displayed than those depicted in standard stimuli of “prototypical” facial emotion expressions. The video scenes or scenarios from films depicting naturalistic facial expressions may be more ecologically valid with regard to expression of emotions and mental states.

Atypical visual processing underlies poor eye contact, and joint attention during social interaction is considered another core characteristic in individuals with ASD (36). Eye contact serves as an important early social function with abilities in recognizing and expressing emotions (37), regulating face-to-face interactions (38, 39) and fostering emerging social skills (40). While ASD individuals may exhibit impairments in facial emotion recognition and abnormalities in visual processing, analyzing their visual processing during facial emotion expression could provide crucial insights into the mechanism of facial emotion recognition impairments in children with ASD. Eye tracking is a valuable objective and quantitative technique in elucidating the underlying visual processing strategies. As eye tracking is noninvasive, with high ecological validity, and it does not require advanced motor responses or any language skills, it is particularly suitable for children with ASD. Previous studies have demonstrated that different eye gaze patterns were expressed in identifying different emotions among TD children. They exhibited different visual attention patterns in relation to the valence of emotions: individuals fixated more on the eyes of negative emotions but on the mouth of positive emotions (41, 42). Yet, eye-tracking studies of children with ASD revealed inconsistent findings (43). The inconsistencies may in part be due to the varied stimuli or tasks used, and their inability to adequately capture their attention. For example, no different eye gaze patterns were found in ASD children using static “prototypical” facial emotion expressions (26, 27, 44), whereas other studies reported a reduced number of fixations or duration of time spent on looking at the faces or eyes’ regions when viewing dynamic stimuli in children with ASD when compared with TD children (28, 45). These discrepant results seem to imply that the static stimuli may not be suitable to engage children in the study and thus were not capable of eliciting information on expected gaze behavior. In addition, the film scene stimuli have been demonstrated to be a valuable form of stimuli to quantify the emotion recognition skills that can distinguish high-functioning children with ASD from the matched controls (32, 33). Unfortunately, to our knowledge, there is a lack of study investigating the different social visual attention patterns in children with ASD using ecologically valid emotion stimuli. An eye tracking study of ASD individuals during presentation of dynamic and naturalistic stimuli could reveal the behavioral and neuropsychological characteristics of children’s recognition strategies of real-life emotions, and allow comparison and reveal the possible differences between ASD and TD children. Therefore, characterizing their eye gaze trajectory may quantitatively reveal the pattern and severity of expression recognition deficits, which could be used in the accurate and effective assessment of children with mild or moderate ASD.

As discussed above, the current study aimed to assess the profile of intelligence and the ability of facial emotion recognition in children with mild or moderate ASD in urban kindergartens of Xi’an, China. GDS-C and eye tracking were used to describe the intelligence profile and eye movement during emotion recognition, and to correlate intelligence with eye movement characteristics, as an attempt to provide theoretical basis for educational decision for children with mild or moderate ASD. Findings could also contribute to a more effective diagnosis for children with mild-to-moderate ASD using eye movement technique in the clinical setting.

## Material and Methods

### Participants

The study was carried out in 12 urban kindergartens of Xi’an city from December 2017 to January 2018. Thirty-six children were diagnosed with ASD among a total of 5,178 kindergarten children by practicing pediatricians who were experienced in children developmental behavior based on the *Diagnostic and Statistical Manual of Mental Disorders-V* (DSM-V) criteria (1) and Autism Behavior Checklist (ABC) (7). All ASD children were subsequently assessed by using the Childhood Autism Rating Scale (CARS), and 21 who were diagnosed with mild-to-moderate ASD (17 males and 4 females) based on CARS were recruited for the present study. The 21 ASD children were of ages from 46 to 83 months (M = 61.10 months, SD = 11.37 months). Twenty-one TD children were matched for birthday (within 6 months) and gender, and they were recruited from same class and kindergartens. The chronological age of the TD group was 45.83–80.67 months (M = 59.31 months, SD = 10.79 months). There were no statistically significant differences in the age of the two groups (*t* = 0.525, *P* = 0.602). All TD children had no psychiatric diagnoses or special educational needs, and none had a family member diagnosed with ASD, as reported by their parents. The CARS was also used to identify the TD children who have normal social and behavioral ability. The basic characteristics of participants were illustrated in **Table 1**. All participants were native Mandarin speakers and passed all screening criteria detailed below. No participants had any visual problems or were in need of corrective eyeglasses. Informed consent was obtained from all parents of participants, and the study was approved by Xi’an Jiaotong University Health Science Center Ethics Committee.

**Table 1:** The basic characteristics of participants.

Characteristics	ASD group	TD group	t	P
Gender (male/female)	17/4	17/4		
Age range (months)	45.97–83.00	45.83–80.67		
Age in months (M ± SD)	61.10 ± 11.37	59.31 ± 10.79	0.53	0.602
ABC score range	36–77	0–10		
CARS score range	25–35	15		

### The Griffiths Mental Development Scales for China

The intelligence profile of each participant was assessed using the GDS-C (3–8 years), administered by a certificated developmental pediatrician. The Griffiths’ test consists of six subscales, including Locomotor subscale (gross motor development such as balance and coordination of movements), Personal-Social subscale (self-skills of daily activities, social skills of interaction and communication), Language subscale (receptive and expressive language), Eye–Hand Coordination subscale (fine movements including drawing and writing), Performance subscale (visuospatial skills, imitation and processing speed), and Practical Reasoning scales (logical thinking). The combination of all six subscale scores was assigned as the General Quotient (GQ).

Percentile rank (PR) was calculated for all neurodevelopmental scores in order to compare the intelligence level within the same age. Neurodevelopmental outcome was classified as normal (PR ≥ 15), mildly delayed (2.5 ≤ PR < 15), or severely delayed (PR < 2.5).

Both ASD and TD participants were assessed using GDS-C and CARS by two developmental behavior pediatricians, and every assessment was under the supervision of an MD/PhD professor in kindergartens. The CARS and parents’ report content scoring in Griffiths test were based on information gathered from teachers as well as from field observations.

### Facial Emotion Expression Tasks

#### Materials

**Figure 1:**
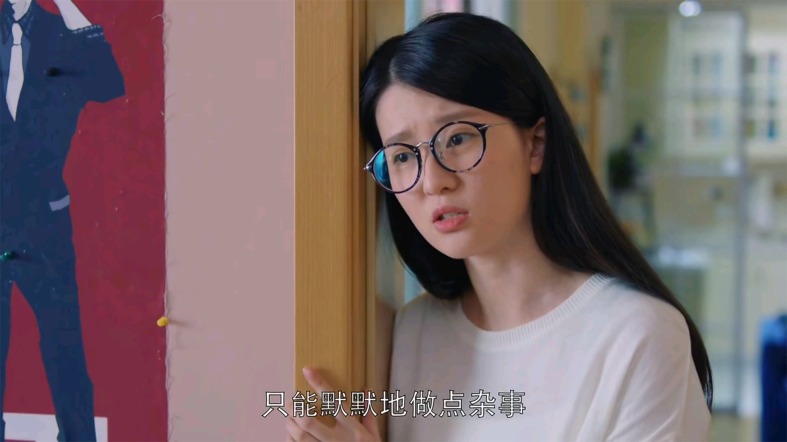
An example of a film scene from the experiment task.

Twenty short film scenes (3.48–6.14s long, M = 4.27 s, SD = 1.25 s) were selected from CASIA Chinese Natural Emotional Audio-Visual Database (CHEAVD) (46). This database provides basic Chinese emotion expression resources for research on multimodal and multimedia interactions (47). All short scenes in this database have been labeled with its corresponding emotion expression. The selected scenes involved socio-emotional interaction ranging from 1 to 2 characters, and the expression of two basic emotions and mental states (happiness and sadness). In each scene, a frontal face protagonist was identified on the center of screen and their emotion or mental state during scenes had been labeled. The selected scenes involved 11 samples with only women characters, 5 samples with only man characters and 4 samples of two characters’ communication. The scenes were selected from TV drama made at least five years’ ago from now and played by nonfamous actors/actresses to decrease the probability that participants had seen them or were familiar with the actors/actresses. **Figure 1** depicts, for example, showing a scene with a sadness young lady leaning against the wall, complaining that she had tons of work to do at the end of the year and will be fired soon.

#### Apparatus

To collect and quantify the eye-gaze data, a contactless eye-tracker SensoMotoric Instruments (SMI) RED 250 (SMI Technology, Germany) was used. The eye-tracker system is composed of an eye tracker hardware, a 22-inch monitor with a resolution of 1280 × 1024 pixels, and a stimuli presentation and control computer. The sampling rate was set at 120 Hz with an accuracy of 0.4°. The freedom of head movement was 40 cm × 20 cm at 70 cm distance. The experiment was prepared and presented in SMI Experiment Center^™^. Fixation detection was performed through the SMI BeGaze^™^ using Dispersion-Threshold (DT) algorithm. Eye-gaze data was recorded online with the software of Experiment Center 2.0 and analyzed offline with SMI BeGaze analysis software.

#### Procedure

Participants were tested individually in a quiet and well-lit room in the childcare center, which had no external light stimulus. Before the start of the testing session, participants were trained to get familiar with positive and negative emotions. Typical questions, such as how do you feel when eating your favorite food/when your pet ills, were asked to test if participants understand the two emotions. Besides, pictures and videos that describe various emotions were played by a tablet (Samsung ST800), and participants were instructed to choose a card, each of which correspond to a different emotion. Participants were praised for correct responses. The purpose of this training is to familiarize the participants with the emotions.

After completing the training session, participants were adjusted to sit 60–80 cm from the eye-tracking monitor. The experimenter first calibrated the participant’s eye movements with the built-in five-point SMI calibration procedure, in which the participant had to track a moving dot across the screen with their eyes. Recalibration was required if the calibration results were poor. After calibration, each participant passively watched the video stimuli and was told to concentrate on the speech and actions of the actor/actress. Videos could be played for multiple times if the participant could not understand what was shown in it. After watching the videos, participants were asked to choose a card, which corresponds to the emotion of the actor/actress. The cards were shuffled while the participant watched the videos to prevent them from choosing the same one without consideration. The testing session included 20 videos, which were played randomly.

#### Data Analysis

**Figure 2:**
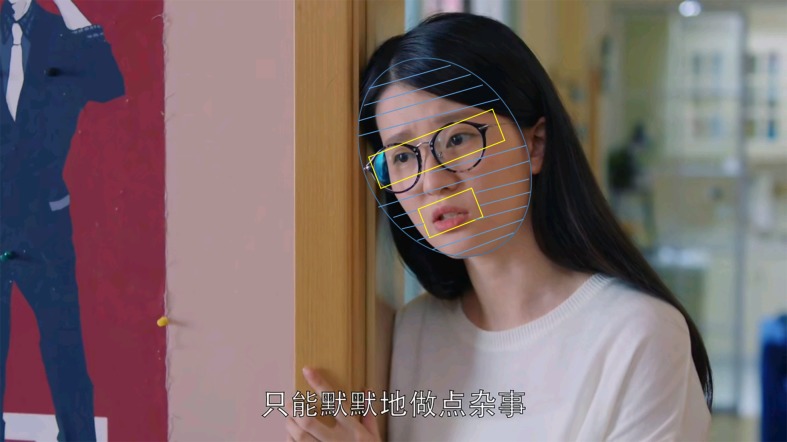
Example of film scene and the delimiting of each area of interest (AOI) boundaries for eye tracking analysis.

To avoid possible effect of gender, multicharacters model, the scenes expressed by only woman characters were chosen to further analysis, and thus 10 scenes were used in eye gaze analysis. In addition, to quantify visual gaze patterns, three areas of interest (AOIs) (i.e., eye, mouth, and face excluding eyes and mouth) were selected for further analysis (see **Figure 2**).

The eye region included, in the horizontal direction, the area ranging from the leftmost corner of the left eye to the rightmost corner of the right eye, and in the vertical direction, the area between the lower side of the eyebrow and the middle nose. The mouth region included, in the horizontal direction, the area between the upmost lip to the bottommost lip, and in the vertical direction, the area ranging from the leftmost lip corner to the rightmost lip corner. The face region included the face except the rest of the eyes and mouth. When identifying emotions, the key regions included the eyes, mouth, and face (excluding the eyes and mouth), which constitute the overall AOI. In addition, the visual fixation of body gesture of protagonist compared to face was also analyzed.

In percent study, three eye-tracking parameters were calculated. The first parameter was the fixation duration time (FDT), defined as the total time of fixation lasting more than 100 ms, reflected the time taken by the participants to think. The second parameter was fixation count (FC), which evaluated the length of fixation lasting more than 100 ms, reflected the absolute attention played by the participants during the process of the experiment. The third parameter was the proportion of AOIs in the overall videos (PA), which was considered as the proportion of FDT and FC in AOI in the whole videos, reflected the degree of dispersion of the subject while watching. The minimum fixation duration was chosen as eye-tracking data of 100 ms (48). Since the videos were of unequal durations, all parameters were normalized by using proportion of AOI in the total videos times. Average fixation duration percentage and FC percentage were calculated across the presentations of each emotion for each region. All data analyses were done in MATLAB and statistical tests (a level = .05 unless otherwise stated) were calculated using Statistical Product and Service Solutions (SPSS) (SPSS Inc., Chicago, IL). Finally, the correlation between the accuracy of emotion recognition, the scores of GDS-C and eye-gaze parameters were analyzed by utilizing Spearman’s rank correlations.

### Statistical Analysis

Matched case–control design was used in the study, in which each ASD-TD children pair was matched by age (within 6 months) and gender, and both were originated from the same class. As GQ and subscales scores of GDS-C and accuracies of emotion recognition are independent and normally distributed with homogeneous variance, paired-sample *t*-tests were used to evaluate possible group difference in GQ, subscales scores of GDS-C, and accuracies of emotion recognition. A two-way repeated-measures ANOVA (two groups × two emotions) was conducted to compare the different patterns of eye-gaze model between children with ASD and TD children. Two-way repeated-measures Multivariate analysis of variance (MANOVA) were used to compare the different eye-gaze patterns in the three AOIs of the face. Pearson correlation analysis was used to evaluate the correlation among Griffiths Scale, eye movement modes, and the ability of emotion recognition. All statistical analyses were performed using SPSS Version 17.0 (IBM Corp). An alpha level of *p* = .05 was used to determine for significant results.

## Results

### Neurodevelopmental Assessment

The paired sample *t*-tests revealed that there was a significant difference in the GQ score, which is 65.78 ± 13.78 for the ASD group and 76.70 ± 12.30 for the TD group (*t* = −3.711, *p* = 0.01). On the subscale scores, except for Eye-Hand Coordination subscale, there were statistically significant differences between two groups in the Locomotor, Personal-Social, Language, Performance, and Practical Reasoning subscales and GQ. The subscales and GQ scores of the GDS-C are summarized in **Table 2**.

**Table 2:** The subscale and GQ scores between ASD group and TD group in the GDS-C.

The subscale and GQ scores	ASD group *n* = 21 (M ± SD)	TD group *n* = 21 (M ± SD)	t	P
Locomotor	63.05 ± 13.81	76.10 ± 12.61	−3.659	0.002*
Personal-Social	71.60 ± 16.20	80.76 ± 8.04	−2.851	0.010*
Language	67.71 ± 18.05	80.76 ± 12.42	−2.913	0.009*
Eye–Hand Coordination	57.69 ± 22.11	65.43 ± 21.43	−1.693	0.106
Performance	63.24 ± 17.83	73.81 ± 15.44	−2.977	0.007*
Practical Reasoning	69.24 ± 16.52	83.33 ± 12.54	−3.884	0.001*
GQ	65.78 ± 13.78	76.70 ± 12.30	−3.711	0.001*

GDS-C, Griffiths Mental Development Scales for China; GQ, General Quotient; ASD, Autism spectrum disorder; TD, typical developing.

*p<0.05.

By comparing the structural mode of each subscale (3–8 years) scores between the two groups, we found statistical differences in the following areas including physical strength, gross body coordination, gross visual motor coordination, social skills for communication, receptive language, basic conception, semantic reasoning, expressive language, imitation, social reasoning, sequence reasoning, and conception. The other structural mode scores between the two groups didn’t have statistical differences. The detailed statistical difference structural mode of each subscale scores between ASD group and TD group are shown in **Table 3**.

**Table 3:** The statistical difference structural mode of each GDS-C subscale scores between ASD group and TD group.

Subscale	Structural mode	ASD *n* = 21 (M ± SD)	TD *n* = 21 (M ± SD)	t	P
Locomotor	Physical strength	4.48 ± 2.522	6.48 ± 2.182	−2.898	0.009*
	Gross body coordination	3.43 ± 4.154	6.38 ± 5.005	−2.444	0.024*
	Gross visual motor coordination	3.33 ± 2.852	6.10 ± 3.974	−2.711	0.013*
Personal-Social	Social skills for communication	4.10 ± 2.406	5.62 ± 1.359	−3.344	0.003*
Language	Receptive language	15.24 ± 9.088	21.81 ± 9.119	−2.502	0.021*
	Basic conception	15.24 ± 9.088	21.81 ± 9.119	−2.502	0.021*
	Semantic reasoning	14.86 ± 8.284	20.67 ± 8.422	−2.343	0.030*
	Expressive language	40.19 ± 15.961	49.14 ± 16.206	−2.124	0.046*
Performance	Imitation	5.90 ± 2.931	7.90 ± 3.064	−2.789	0.011*
Practical Reasoning	Social reasoning	4.00 ± 2.098	5.81 ± 2.089	−3.189	0.005*
	Sequence reasoning	3.33 ± 4.211	6.00 ± 5.477	−2.87	0.009*
	Conception	15.71 ± 5.226	18.86 ± 5.237	−2.271	0.034*

GDS-C, Griffiths Mental Development Scales for China; ASD, autism spectrum disorder; TD, typical developmental.

*p < 0.05.

### Accuracy of Emotion Recognition

**Figure 3:**
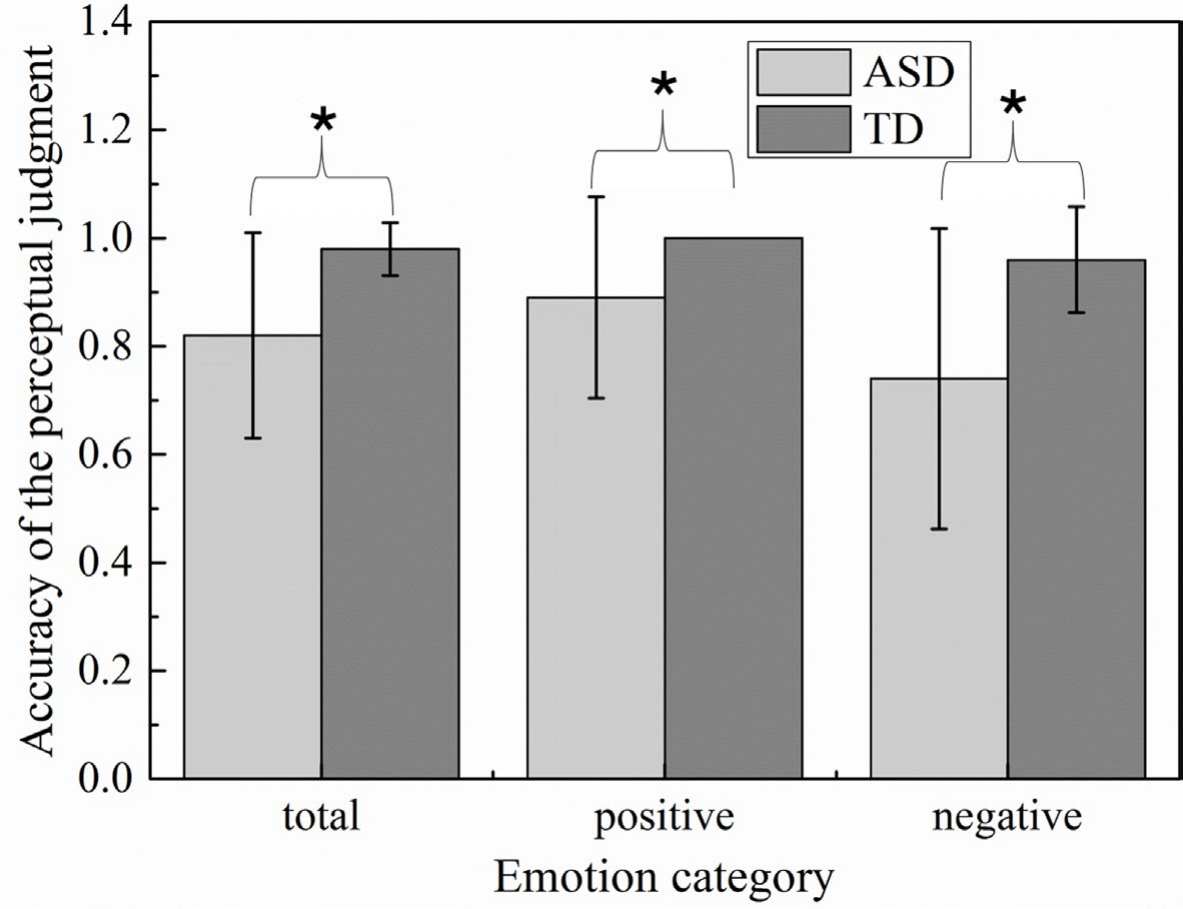
Emotion accuracy of the perceptual judgment in children with autism spectrum disorder (ASD) and typically developing (TD) groups.

To examine whether groups were different in accuracy of emotion recognition, accuracy variable was generated by averaging the identification accuracy across all scenes within each emotion. The details of identification accuracy in multimodal emotion perception were described in **Figure 3**. The perceptual accuracies of children with ASD were significantly less than those of TD children on both happiness emotion (*t* = −2.52, *p* = 0.021) and sadness emotion (*t* = −3.444, *p* = 0.003). Among which, the performance of happiness emotion was significantly better than those of sadness emotion in children with ASD (*t* = 2.22, *p* = 0.04). The children with ASD were seemingly easier to distinguish happiness than sadness emotion. In addition, to evaluate whether severity of the social deficits measured by the Griffiths Development Scales (GDS) was related to accuracy to recognize emotions, Pearson correlations were calculated between the scores of Developmental Quotient, Personal-Social subscale, and the mean identification accuracy on each emotion. Significant relationships were found only in the ASD group between accuracy and the GDS Developmental Quotient in total emotion(*r* = 0.581, *p* = 0.009) and sadness emotion (*r* = 0.531, *p* = 0.019), indicating that children with high Developmental Quotient T-scores (i.e., less severe deficits) made less emotion recognition errors. In addition, the significant correlations were found between Personal-Social subscale score and accuracy on total emotion (*r* = 0.479, *p* < 0.05) and sadness emotion (*r* = 0.421, *p* < 0.05).

### Eye-Gaze Analysis

#### Analysis of the Overall Videos

**Figure 4:**
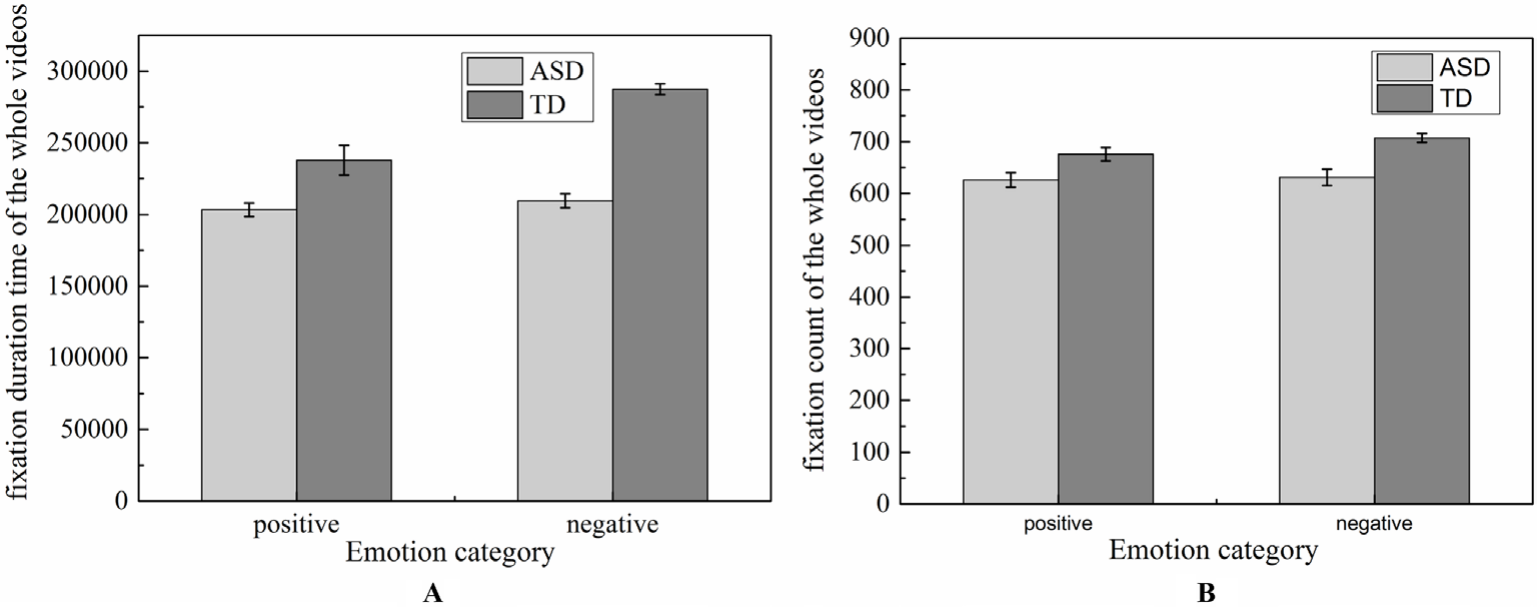
Distribution of fixation duration time and fixation count of the whole videos.

The total numbers of FC and the total FDTs spent on the whole video screen in ASD group and TD group were shown in **Figure 4**. To determine whether the groups differed on positive and negative emotion, the two-way ANOVA (two groups × two emotions) was conducted on FDT and FC, respectively. No significant effects were found in emotion [F (1,124) = 4.90, *p* = 0.57], group [F (1,124) = 5.832, *p* = 0.52], and effect interaction [F (1,124) = 0.65, *p* = 0.949) for FDT. Similarity results were also found in the statistical analysis for FC [group: F (1,124) = 4.53, *p* = 0.542; emotion: F (1,124) = 6.26, *p* = 0.975]. These results implied that there were similar attention to whole video in children with ASD and TD when they watched the experiment scenes.

#### Ration of Face Expression and Body Gesture

**Figure 5:**
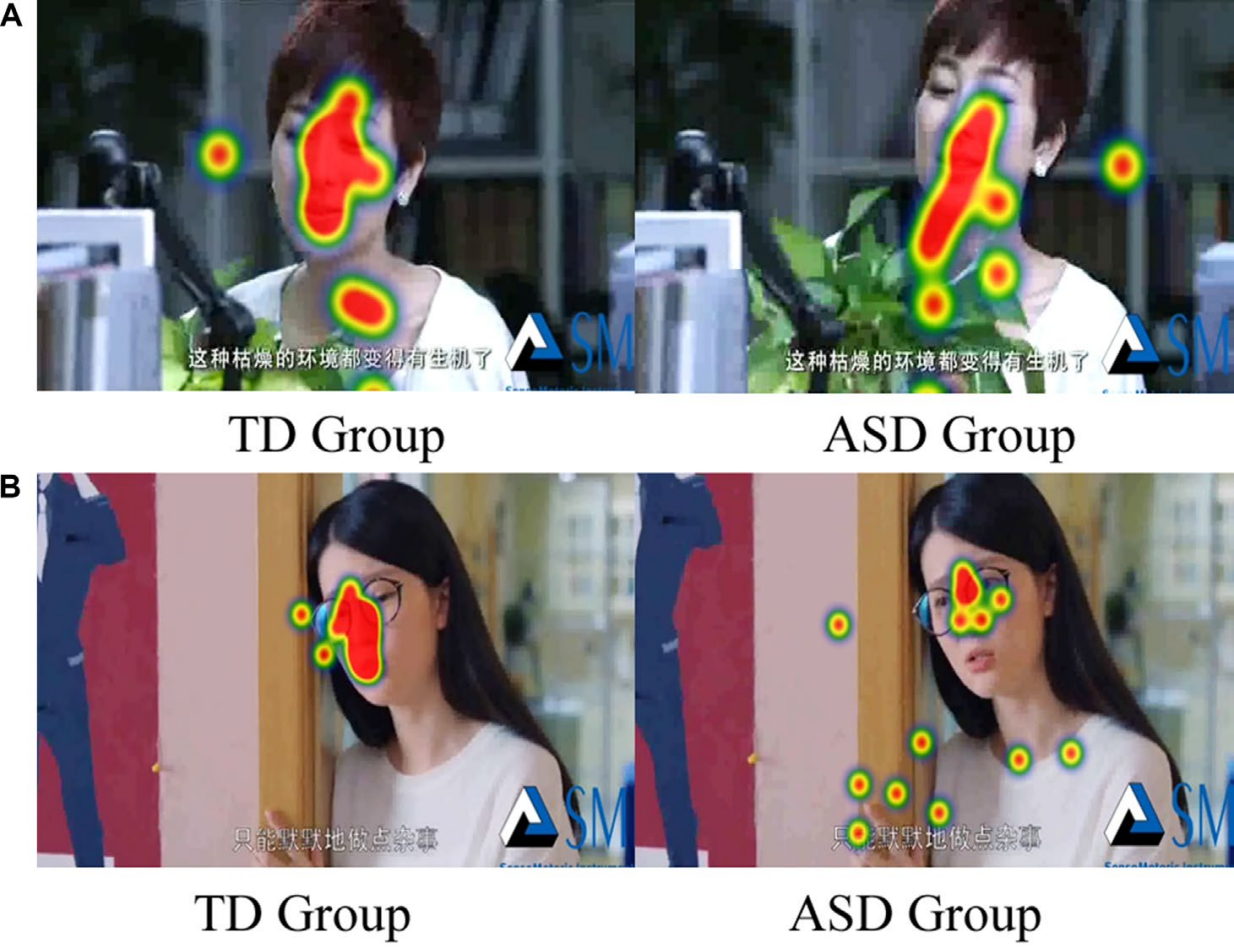
Example of heat map in each emotion expression (**A**: Happiness; **B**: Sadness).

However, when we divided the scenes into face area and body area, the two-way ANOVA (Group* Emotion) on FDT ratio of face and body yielded an overall main effect for group [F (1, 36) = 17.034, *p* < 0.01], but there was no significant difference for emotion [F (1, 36) = 0.101, *p* = 0.752] and interaction effect [F (1, 36) = 0.744, *p* = 0.394].The duration of fixation time on face region in ASD group (M = 0.757, SD = 0.047) was overall significantly less compared to the duration of fixation time in the TD group (M = 0.482, SD = 0.047).The two-way ANOVA (Group* Emotion) on ratio of face and body on FT also found similar results. There was an overall main effect for group [F (1, 36) = 12.520, *p* < 0.01], but there was no significant difference for emotion [F (1, 36) = 2.380, *p* = 0.132] and interaction effect [F (1, 36) = 0.113, *p* = 0.132].The number of FCs on face region in ASD group (M = 0.437, SD = 0.47) was overall significantly less compared to the duration of fixation time in the TD group (M = 0.670, SD = 0.47). In addition, as illustrated in **Figure 5**, the heat maps were also shown the same tendency of this attention distribution in ASD group and TD group. The children with ASD seemed to pay more attention to the neck, breast, and hands of the actor, rather than to face of the actor.

#### Analysis of the Three Areas of Interest of the Face

**Figure 6:**
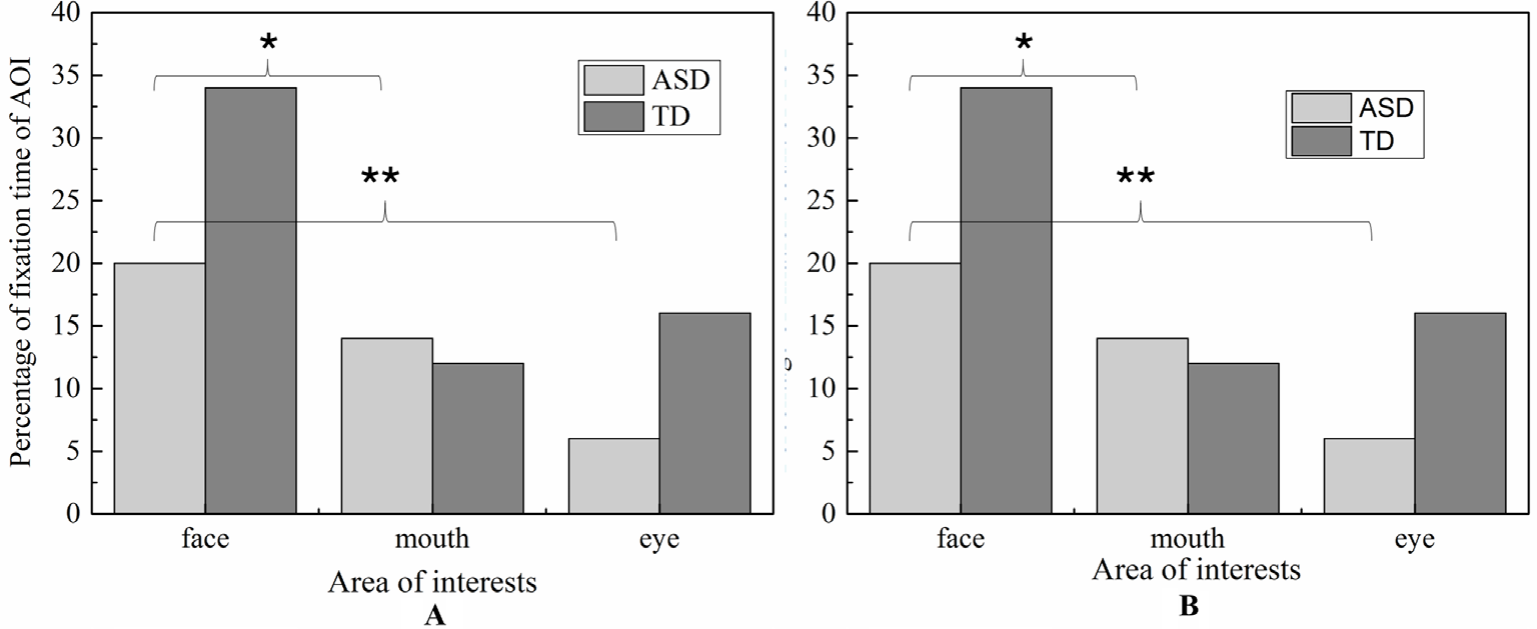
Percentage of fixation duration time and fixation count in each AOI with whole scene on happiness emotion expression.

**Figure 7:**
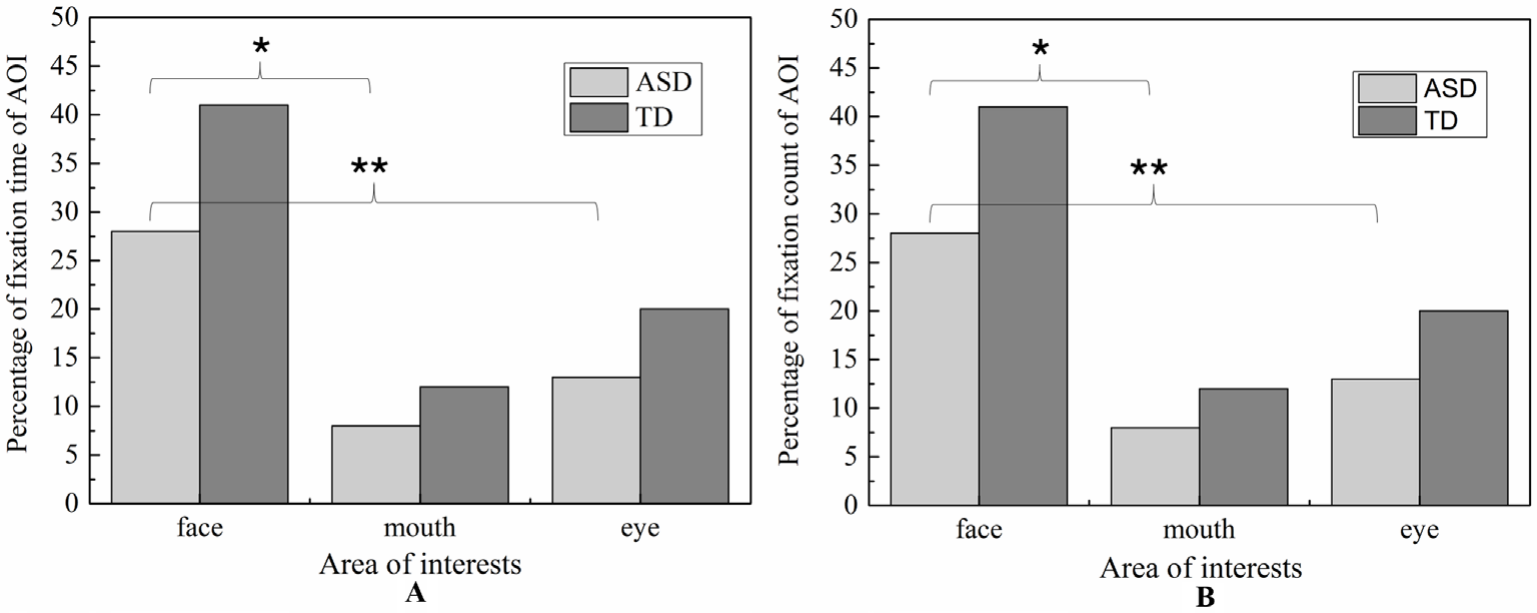
Percentage of fixation duration time and fixation count in each AOI with whole scene on sadness emotion expression.

Due to its centrality in emotional expression, we emphatically analyzed the eye-gaze patterns in face emotion expression. The values of FDT and FT of each AOI in each emotion were illustrated in **Figure 6** and **Figure 7**, respectively. To determine whether the groups differed on emotion (happiness and sadness) and AOI (face, mouth, and eye) in FDT and FC, two MANOVA analyses with repeated measures were conducted, with AOI (face, eye, mouth), and emotion (happiness, sadness) as within subject variables, and group (ASD, TD) as the between-subject variable.

##### Fixation Duration Time

The MANOVA yielded a significant main effect for group [F (1,119) = 8.897, *p* < 0.01]. The duration of fixation time in ASD group (M = 667.187, SD = 42.891) was overall significantly less compared to the duration of fixation time in the TD group (M = 839.116, SD = 38.508). Meanwhile, the main effect of region was significant [F (2.238) = 42.174, *p* < 0.01], and was also significant effect for emotion [F 1,119) = 14.105, *p* < 0.01], which was driven by significant interaction between effect and AOI [F (2,238) = 4.433, *p* < 0.05]. However, there was no significant interaction effect on the three-way interaction between group, AOI, and emotion [F (2,238) = 1.902, *p* = 0.115] and interaction between emotion and group [F (1,119) = 0.005, *p* = 0.944].

In order to explore the those effects, a simple main effect analysis was conducted with emotion as within subject variable and group as the between-subject variable was conducted for average FDT in each AOIs, respectively.

For mouth region, the main effect of emotion was not significant (*p* = 0.752) and the main effect of group was also not significant (*p* = 0.901). Therefore, the fixation time of mouth region had no significant differences in ASD and TD children for each emotion expression.

For eye region, significant effects were identified for group [F (2.238) = 42.174, *p* < 0.01] and emotion [F (1,119) = 14.105, *p* < 0.01]. We then explored the simple effects of group (adjusting for multiple comparisons using Bonferroni) in each emotion separately. The results showed that for both emotion expressions, the fixation times spent on eye region were significantly decreased in ASD group compared to TD group. In order to explore the sample emotion effect, we compared the pairs of emotions in each group and found that both ASD and TD children had more FDT on negative emotion than positive emotion in the eye region [F (1,119) = 14.11, *p* < 0.01).

**Figure 8:**
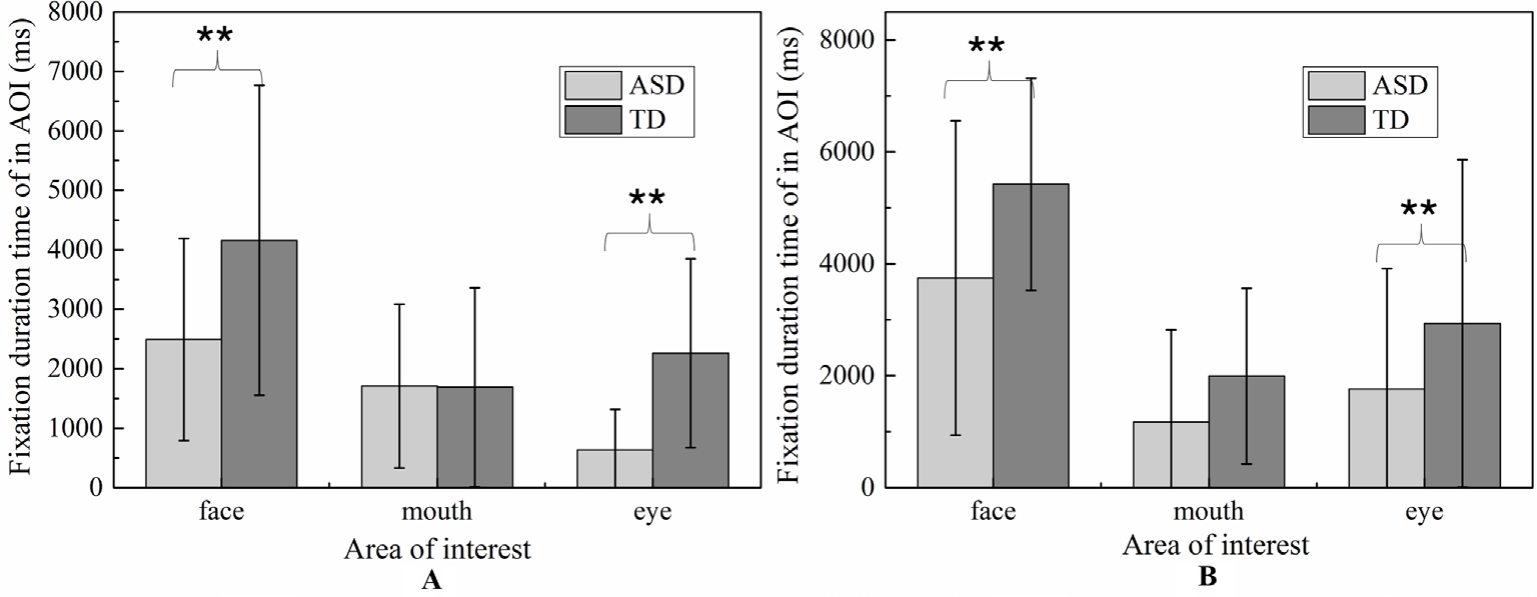
The total fixation duration time of AOI in each emotion expression (**A**: Happiness; **B**: Sadness).

For face region, the significant effect of emotion was found (*p* < 0.01), and *post hoc* comparisons revealed both ASD and TD children had more FDT on negative emotion than positive emotion in the face region. As illustrated in **Figure 8**, the ASD group displays a tendency to look less at the face region than the control group in each emotion. However, probably due to the small sample sizes, the statistical power was too low to detect a group effect.

##### Fixation Count

**Figure 9:**
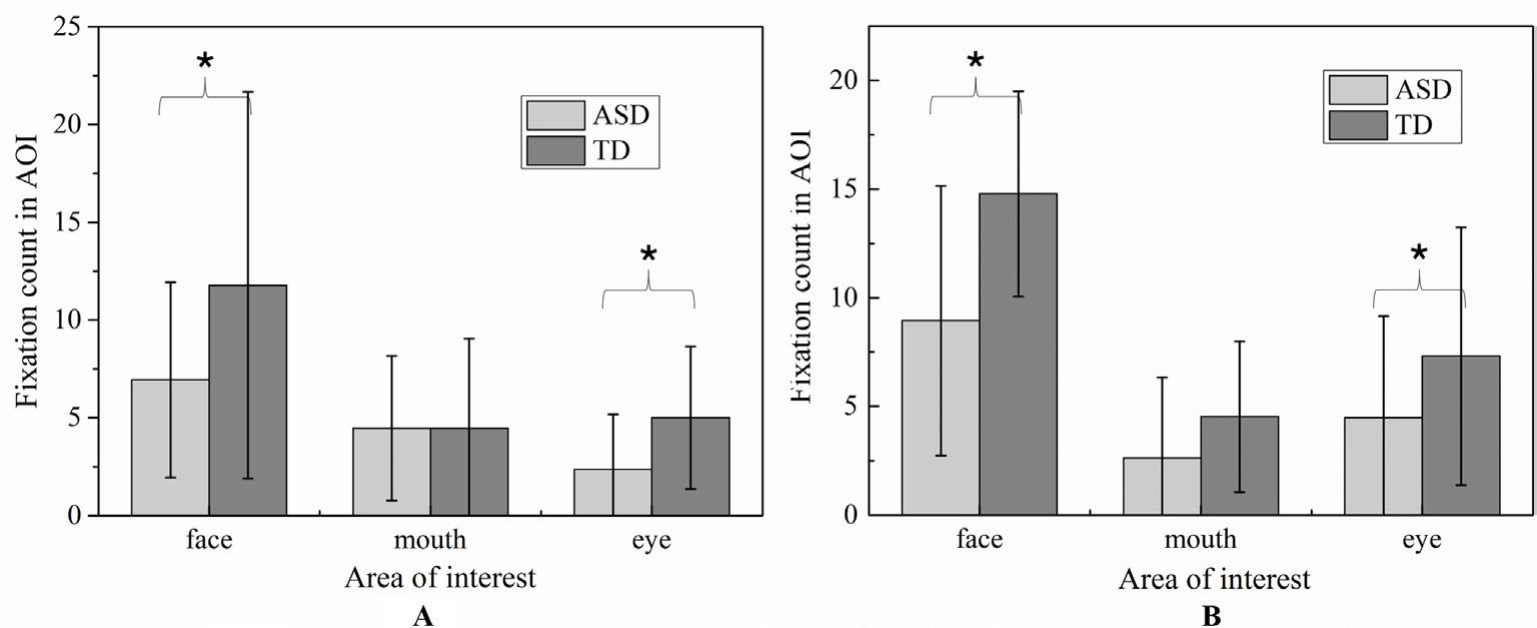
The total fixation count of AOI in each emotion expression (**A**: Happiness; **B**: Sadness).

Similar to FDT, the MANOVA yielded a significant main effect for group: (F (1,107) = 5.800, *p* < 0.05). As illustrated in **Figure 9**, the number of FCs in ASD group (M = 1.715, SD = 0.14) were overall significantly less compared to the FCs in the TD group (M = 2.154, SD = 0.122). We also found the main effect of AOIs (F (2, 214) = 44.285, *p* < 0.01), and emotion (F (1,107) = 5.620, *p* < 0.01), which was driven by significant region*emotion interaction (F (2, 214) = 4.406, *p* < 0.05). But no significant of interaction effect on the three-way interaction between group, AOI and emotion (F (2,238) = 1.902, *p* = 0.115), interaction between AOIs and group, and interaction between emotion and group (F (1,119) = 0.005, *p* = 0.944). Then, the repeated-measures ANOVAs were performed to examine group differences and interactions for each region. The Huynh-Feldt correction was used to adjust for sphericity violations when necessary.

For mouth region, no group and emotion differences were found. For other region, significant effects were identified for group (eye, F (1,107) = 5.800, *p* < 0.05, and face, F (1,107) = 5.800, *p* < 0.05) and emotion (eye, F (1.107) = 5.620, *p* <0.05, and face, F (1,107) = 5.620, *p* <0.05).As evidence in Fig.6, pairwise t-test revealed ASD children had less FCs on eyes and face region than TD children in each emotion condition. Both ASD and TD children had more FCs on negative emotion than positive emotion in the face region.

#### Correlation Analysis

##### Correlations Between Eye Gaze Pattern and Emotion Recognition

To examine whether eye gaze pattern was related to accuracy of recognize emotions, Pearson correlations were calculated between accuracy of emotion recognition and the percentage of FDT and FCs on the whole face region and each AOIs. In the ASD group, significant relationships was found between accuracy of recognize emotions and the percentage of FDT on whole face region (r = 0.702, *p* = 0.001), the percentage of FCs on whole face region (r = 0.492, *p* = 0.032). These correlations indicated that looking longer and more attention at the face region was related to more accurate detections of emotion. However, there were no significant correlations were found in AOIs.

##### Correlations Between Eye Gaze Pattern and the Severity of Development Deficits

To examine whether eye gaze pattern was related to the severity of development deficits in children with ASD, Pearson correlations were calculated between GQ, Personal-Social subscale score and the percentage of FDT and FCs on the whole face region and each AOIs. The percentage of FDT and FCs on whole face region were both moderately positively associated with GQ (r = 0.619, *p* = 0.005 and r = 0.606, *p* = 0.006) in sadness emotion. The percentage of FDT and FCs on whole face region were both moderately positively associated with Personal-Social subscale score (*r* = 0.577, *p* = 0.010 and r = 0.503, *p* = 0.013). However, no significant correlations were found in AOIs.

## Discussion

The present study examined the possible differences in mental development, recognition of emotions, and eye-gaze pattern between children with mild or moderate ASD who were attending regular kindergarten, in comparison with the TD children using GDS-C score and ecologically valid facial expressions task. The relationship among these measures and severity of social impairments in ASD measured by the GDS-C were also explored.

Children with ASD had lower mental development scores in the Locomotor, Personal-Social, Language, Performance, and Practical Reasoning subscales than their TD peers. Moreover, ASD children also showed emotion recognition deficits in facial expressions with naturalized scenes even with contextual vocal expressions integrated. The deficits were significantly correlated with the ability of child interaction and development quotient in ASD group. Eye-gaze analysis also revealed that children with ASD had atypical eye-gaze pattern when compared to TD children in facial emotion expression task. Children with ASD had reduced visual attention to the facial expression, especially for eye regions. Our findings shed light on the deficits of children with ASD to conduct naturalize multimodal emotion recognition, and on the nature of their atypical eye-gaze pattern in terms of emotion recognition.

The GDS allows examination of the main aspects of a child’s development, namely, physical, cognitive, social, and emotional. It is also useful for evaluating the developmental deficits in individuals with neurodevelopmental disorders. In the present study, the preschool ASD children showed lower intelligence scores in the Locomotor, Personal-Social, Language, Performance, and Practical Reasoning subscales than the typical developmental peers, despite the fact that they were attending normal kindergartens. These findings are consistent with Pan (49) and Hedvall’s researches (50), in which a weaker intelligence profile in ASD children than TD peers was also reported. Although children with ASD in the present study were able to communicate with others in simple sentences and generally could cope with their daily life in the kindergartens, they still showed weaker ability in Personal-Social subscale because of the insufficient social and communication skills. Social deficits were considered by Kanner to be central to the pathogenesis of ASD (51). Plenty studies have shown that ASD children lack the abilities in social reciprocity and in developing meaningful relationships on the basis of interpersonal interactions (52–54). Moreover, they also showed reduced ability in understanding, expressing, and forming concepts, achieving semantic reasoning, and imitating behaviors. These deficits are consistent with the core symptoms of ASD, which can be explained by the “broken mirror theory” (55). According to the theory, ASD individuals suffer a hypoactivity of mirror neurons and they are not able to embody in themselves in others’ mental states (intentions, beliefs, expectations, etc)., or the “Theory of Mind.” Moreover, as previous reports suggested, the language function area of an ASD brain overlaps with the mirror neuron system, resulting in concomitant impairment of their language ability (56).

As reported in the literature, more difficulties in emotion recognition based on facial expressions in ecologically valid condition were found for the ASD children, when compared with the TD counterparts, even after controlling for chronological age. Our finding is in line with what was previously reported regarding the difficulties in individuals with ASD on recognizing emotion and mental state from dynamic or multimodel stimuli (15, 57). However, the finding is contradictory to the previous finding from Lacroix’s study (58), in which recognition of facial emotion in ASD participants with similar demographics (high function, age range: 4–8 years, and educated in mainstream schools) was not different from TD individuals. The inconsistent results may be due to the use of different stimuli type: the static “prototypical” stimuli in Lacroix’s study vs. naturalistic film stimuli in the current study. In fact, the discrepant findings reported in the literature could be explained by three possible reasons: 1) stimulation intensity, 2) dynamic expression, and 3) multimodal integration. The prototypical stimuli mostly presented the maximum intensity expression of each emotion and video clips showed smooth transition of each emotion during the whole video, thus that may be the soft expression. Children with ASD are limited to implicit expressions with low intensity levels (59) and that may cause the children with ASD to have poor performance in video clips task. Further studies should investigate the intensity effect on sensitivity for recognition of film emotion expressions in individuals with ASD. Moreover, previous study reported there have specific deficits in processing of dynamic stimuli for ASD (60), noted that all of our stimuli used video clips present by dynamic stimuli, our stimuli may be more challenging to children with ASD, in comparison to static images. Finally, cross-modal integration seems more difficult for individuals with ASD (57, 61); ecologically valid audiovisual stimuli used in the present study might hamper the ability of children with ASD to recognize emotions and mental states. Further studies should reveal the ability of children with ASD on recognition presenting emotional cues in different channels of ecologically valid condition.

In addition, a moderate correlation was found between emotion recognition ability and mental development of children with ASD, especially during social personal interaction. ASD individuals who had poor social personal interaction also showed lower accuracy of facial emotion recognition, particularly in happiness and sadness emotions. These findings suggest that the altered development of emotion recognition in ASD children may be related to deficits in more complex social functions (62). Research has indicated that for TD children of about 4 years of age, their ability in recognizing happiness, sadness, and anger is maturing (16, 63). This is in line with the current findings regarding TD children who had matured emotion recognition in real-life environment. However, despite the use of only two facial stimuli of happiness and sadness emotions considering participants’ age range, deficits in emotion recognition were still observed in our 3–7-year-old ASD children. The deficits were related to risking the ability to represent and communicate one’s own internal states and feelings, and cause deficits in the self-regulation of behavior and social interactions (64, 65). Taken together, our findings reveal a comprehensive deficit in emotion recognition among our 3–7-year-old children with ASD.

As hypothesized, the ASD children showed atypical visual social attention under a naturalistic environment. First, the children with ASD spent less attention and time looking at the face and more attention to the bodies and other objects, although both groups spent similar attention to the entire video. The present finding is consistent with previous studies using similar stimuli (66, 67) in which 5 year-old children with ASD were found to spend less time looking at faces in comparison to TD children and children with specific language impairment (Geraldine Dawson et al.). With careful analyses of scanning gaze pattern of each stimulus, children with ASD seemed to change attention to face quickly. This might imply that children with ASD had social sustained attention deficits during complex social situations involving eye contact and speech (68, 69). The deficits may correspond to hypo-activation in the face processing cortical system including amygdala, fusiform face area (FFA), superior temporal sulcus (STS), and the occipital face area (70, 71), which might result in increased autonomic arousal and trigger self-regulatory strategies such as gaze aversion. The correlation analysis also indicated that the limited ability in sustaining attention on the face was associated with the diminished ability of emotion recognition, and personal-social interaction and intelligence development deficits. Given that visual social attention plays a crucial role in the development of more sophisticated patterns of the basis for cognitive and social development (72), atypical face processing in ASD individuals could arise from socio-cognitive factors and be exacerbated by the complex visual information conveyed by human faces in social interactions, and further compound by social-cognitive impairments. As a matter of fact, some studies have posited that social-cognitive impairments in ASD individuals could primarily result from a failure to orient and engage attention to socially relevant stimuli such as faces early in life (17, 25, 73, 74).

More specifically, our ASD children exhibited significantly diminished visual social attention in eyes and no-core face regions but no significant difference in mouth region during watching both emotion perceptions. These findings are somewhat consistent with the previous study (30) which reported reduced visual fixations to the eyes region in face familiarity on neutral emotion perception. However, our results regarding the mouth region are inconsistent with Nuske’s, in which TD children were found to fixate more to the mouths of neutral expressions than children with ASD when viewing familiar and unfamiliar faces (30). Because our stimuli were visual with audio signals integrated, and mouth movement in the visual stimuli carried audiovisual information that might help process emotion language information, children with ASD seemed to focus more on the mouth region as a compensatory strategy that might help them to achieve better recognition when facing complex social situations. That may partly explain why our ASD children showed greater accuracy on happiness emotion recognition than sadness condition as the mouth region was core characterization of “happiness” expression, whereas eye region is characterization of “sadness” expression.

Analyzing the entire visual stimuli of the actress, attention distribution pattern of children with ASD seemed to display a “downward laterality” pattern and pay more attention to the area below the eyes, such as the neck, breast, and hands of actress, rather than to the face of actress. Diminished visual social attention in the eye region and the “downward laterality” pattern can be explained using the “eye avoidance hypothesis” (75). The hypothesis postulates that individuals with ASD may present with over-arousal of the amygdala and hyper-physiological arousal in response to social stimuli. As a result, reduced gaze to the eyes in individuals with ASD may be an attempt to self-regulate and mediate the level of threat perceived from the eyes (70, 75). In the present study, ASD children explored the self-regulatory strategies of gaze aversion and consciously avoided actress’s eyes. However, correlation between visual attention and accuracy of emotion recognition was only found for the entire face region, but not found for all AOI areas. That implies that children with ASD utilized a compensatory strategy on the entire face to achieve better recognition when facing complex social situations, and made use of core characteristic to recognize emotion expression, particularly for negative emotion. Future studies should investigate the ability of young children with ASD to modulate their attention in response to different emotion expression systematically varying in their communicative intent.

## Limitations

Some limitations can be identified from the current study. The first limitation relates to the small sample size. This is due to the difficulty associated with identifying the appropriate children participants. There are very few ASD children in mainstream schools. As revealed in a recent survey of three cities of China, only 9 cases of ASD were identified from a total population of 6,240 aged 6–10 years old in mainstream schools in Jilin City, and 35 cases of ASD were identified from 21,420 children in Shenzhen City, and 10 cases of autism were identified from 16,358 children In Jiamusi City (76). In the present study, 36 children diagnosed with ASD were selected from a total of over 5,000 kindergarteners based on formal assessments (DSM-V and ABC). The ASD children were further shortlisted as mild-to-moderate grade based on CARS. As such, interpretation of results has been made with caution. Future studies are suggested to include more homogeneous participants to warrant more representative findings. In addition to the small sample size, gender difference associated with emotion recognition was not examined due to the small number of female ASD participants. The present study started by recruiting unidentified ASD participants from mainstream kindergartens, and thus gender distribution could not be controlled. Previous studies reported contradictory finding on gender difference in adults of ASD (32, 77). Future studies should expand the sample size in order to include sufficient samples to examine gender effect on emotion recognition in ASD children. Since ASD is a heterogeneous condition of varying severity and nature, future studies should employ a larger sample, allowing examination of emotion recognition between subgroups within ASD individuals of different verbal mental age.

Secondly, the present study only examined the first mature basic emotions: happiness and sadness. In order to understand the unique mechanism of facial emotion recognition in children with ASD under naturalistic environment, further studies might include other basic emotions and some complex emotions, especially fear and anger. Deficits in responding fearful expression by ASD children have been documented, which is correlated with amygdala activation (30, 78). If more basic and complex emotion expression is included in future studies, the delay in mental development as well as the deviant pattern of emotion recognition in ASD children could be understood, and that will allow better understanding of facial emotion recognition in children with ASD and cognitive deficits.

Basic parameters of eye tracking including time and frequency of fixation were analyzed in the study. Yet, the scanner pattern of children with ASD appears to be atypical in heat map. Future studies should utilize more adaptive methodologies for eye tracking data analysis such as saccades analysis, iMAP methods, and scanning paths analysis (79, 80), so as to reveal more information about attention shift between facial features and to better understand the facial pattern processing in individuals with ASD. Further study should also combine eye-tracking with neurophysiological measures such as EEG and functional near-infrared spectroscopy (fNIRS) (43), which could provide greater insights in the understanding of the electrophysiological mechanisms being moderated in relation to specific gaze behaviors.

In summary, the current study explored retardation of mental development, deficits in emotion recognition, and atypical eye gaze in children with mild or moderate ASD who were attending regular kindergarten. The emotion recognition deficits were significantly correlated with their ability in social interaction and development quotient in ASD group, as well as atypical eye-gaze pattern. The findings confirm the deficits of ASD children in real-life multimodal of emotion recognition, and exhibiting eye avoidance pattern during emotion recognition. The findings suggest the parents and teachers of mild or moderate ASD children should make informed educational decisions according to their level of mental development. In addition, eye tracking technique might clinically help diagnose children with mild or moderate ASD.

## Ethics Statement

This study was carried out in accordance with the recommendations of consent protocol of Biomedical Research in Xi’an Jiaotong University, Xi’an Jiaotong University Health Science Center Ethics Committee with written informed consent from all subjects. All subjects gave written informed consent in accordance with the Declaration of Helsinki. The protocol was approved by the Xi’an Jiaotong University Health Science Center Ethics Committee.

## Author Contributions

YH and QS contributed equally to this work. YC, LW, and NY designed the project; YH, QS, WH, CT, and HZ performed the experiments and collected the data; YH, QS, and NY analyzed the data; YH, QS, YC, LW, MN, and NY interpreted the results and wrote the manuscript. All authors contributed to manuscript revision, read and approved the submitted version. All authors provide approval for publication of the content and agree to be accountable for all aspects of the work in ensuring that questions related to the accuracy or integrity of any part of the work are appropriately investigated and resolved.

## Funding

This study was jointly supported by a grant from National Natural Science Foundation of China (NSFC 61771461, NSFC 81371900, and U1736202), The Province Natural Foundation of Shaanxi (2009JM4030), The Province Science and Technology Research Projects of Shaanxi (2013SF2-09), Shenzhen Speech Rehabilitation Technology Laboratory and Health and Health Services Research Fund (HHSRF), Shenzhen Fundamental Research Program JCYJ20170413161611534, JCYJ20160429184226930, and KQJSCX20170731163308665.

## Conflict of Interest Statement

The authors declare that the research was conducted in the absence of any commercial or financial relationships that could be construed as a potential conflict of interest.
